# Mechanical thrombectomy failure in anterior and posterior circulation stroke: current results from a high-volume comprehensive center

**DOI:** 10.1007/s10072-024-07881-2

**Published:** 2024-11-23

**Authors:** Alexander Neumann, P. Schildhauer, S. M. Weiler, P. Schramm, H. Schacht, G. Royl, U. Jensen-Kondering

**Affiliations:** 1 Department of Neuroradiology, University Medical Center Schleswig-Holstein, Campus Lübeck, Ratzeburger Allee 160, Lübeck, 23538 Germany; 2 Experimental Psychology Unit, Humanities and Social Sciences, Helmut Schmidt University / University of the Federal Armed Forces Hamburg, Holstenhofweg 85, Hamburg, 22043 Germany; 3 Department of Neurology, Neurovascular Center, University Medical Center Schleswig-Holstein, Campus Lübeck, Ratzeburger Allee 160, Lübeck, 23538 Germany

**Keywords:** Stroke, Large vessel occlusion, Distal medium vessel occlusion, Mechanical thrombectomy failure, Direct aspiration, Stent retriever

## Abstract

**Background:**

Mechanical thrombectomy (MT) is an established therapy for acute ischemic stroke (AIS), but recanalization is not always achieved. Common reasons are inadequate removal at the thrombus site and difficulties with the access route. In order to identify risk factors for MT failure we conducted a retrospective study on a high-volume comprehensive stroke center.

**Methods:**

Evaluation of 552 thrombectomies (2019-23; anterior and posterior circulation, direct aspiration +/- stent retriever *[*SR]). MT failures (= modified Thrombolysis in Cerebral Infarction score 0 or 1) were analyzed for age, sex, pre- and post-MT modified Rankin Scale, bridging intravenous thrombolysis (IVT), occlusion site (anterior / posterior circulation, proximal / distal), the Kaesmacher classification and time trend results.

**Results:**

MT failure occurred in 56 patients (10.1%; median age 76; 53.6% female). Nineteen (33.9%) patients received IVT (*p* = 0.326). Logistic regression analysis did not show a significant association of age, sex or occlusion site with MT failure (*p* = 0.165, *p* = 0.738, *p* = 0.838). Distal MT generally demonstrated lower success rates (*p* < 0.01). According to the Kaesmacher classification SR failure was the most frequent cause of MT failure (category 2B: 48%, *p* < 0.001). Time trend analysis suggests improving recanalization rates in the further course (4 times in year-on-year comparison; *p* < 0.01).

**Conclusion:**

MT failure occurs in AIS treatment, even in high-volume centers and occurs more frequently in distal occlusions. Improvements in device technology, particularly SR, and ongoing refinements in access route selection offer the prospect of better outcomes in the future.

## Background

In acute ischemic stroke (AIS), mechanical thrombectomy (MT) using aspiration techniques in combination with stent retrievers is established for recanalization of large vessel occlusions (LVO) in both the anterior and posterior circulation [1–6]. The frequency of MT is increasing overall, particularly with regard to the recent positive randomized controlled trial results even in large infarct cores and later time windows [7–11]. In addition, the age range for this endovascular procedure has grown in recent years, with the additional and primarily self-evident inclusion of children and adolescents as well as old and very old patients despite recommended careful selection of them [12, 13]. The spectrum is also extended by increasing evidence for MT of distal arteries [14–17]. MT failure (= modified Thrombolysis in Cerebral Infarction score [mTICI] 0 or 1 respectively 0-2a) is generally a known caveat in the treatment of AIS and has already been reported in several studies with frequencies between 10.6 and 26.1% [18, 19, 20–26; Table 1]. Common reasons for MT failure are a missing device breakdown during the thrombus removal itself and inadequate cervical or intracranial access routes [19, 23, 25, 27]. These findings raise the question of more individualized planning and implementation of interventional stroke therapy to avoid MT failure, especially with a focus more on the specific thrombus optimized devices and potential bailout strategies to control anatomical problems [28–30]. While taking this into account, Kaesmacher et al. developed a classification for MT failure in particular with stent retrievers considering non-technical or technical reasons [19; Table 2].

**Table 1:** Previous studies addressing mechanical thrombectomy failure [16, 17, 19–25]

Authors, year of publication	Number of patients	Study design	Study period	MT failure definition (mTICI)	MT failure rate	Technique	Anterior circulation	Posterior circulation	Inclusion of distal vessel occlusions (up to)
Our study	552	Single center	2019–2023	0/1	10.1%	DA + SR	+	+	M3, P2
Lajthia et al., 2023	1010	Single center	2013–2021	0-2a	11.9%	DA + SR	+	+	M4
Marnat et al., 2022	5076	Multicenter (*n* = 8)	2015–2020	0-2a	12.4%	DA + SR	+	-	no
Flottmann et al., 2021	2211	Multicenter (*n* = 25)	2015–2028	0-2a	16.8%	DA + SR	+	-	no
Weyland et al., 2021	218	Single center	2009–2019	0/1	13.8%	DA + SR	-	+	no
Heider et al., 2020	596	Single center	2014–2018	0-2a	16.8%	DA + SR	+	-	no
Goda et al., 2019	119	Single center	2015–2019	0-2a	26.1%	DA + SR	+	-	M3
Leischner et al., 2019	648	Single center	2010–2017	0/1	11.0%	DA + SR	+	-	no
Kaesmacher et al., 2018	592	Single center	2012–2017	0/1	10.6%	SR	+	+	no
Gascou et al., 2014	144	Single center	2009–2011	0-2a	13.9%	SR	+	+	n.a.

Legend: DA - direct aspiration, MT– mechanical thrombectomy, mTICI - modified thrombolysis in cerebral infarction scale, n.a. - not applicable, SR - stent retriever

**Table 2:** Known classification of mechanical thrombectomy failure reasons as published by Kaesmacher et al. [19]. The changes we have added are highlighted in italics

Category	Classification system for mechanical thrombectomy failure
1	**Technical reasons: Target occlusion not reached** 1 A: Intracranial target occlusion was not reached due to marked cervical vessel tortuosity including twisted, looped, or kinked vessels. The proximal cervical vessels were successfully catheterized. 1B: Target occlusion was not reached owing to failed catheterization of proximal supra-aortic vessels. The proximal cervical vessels were not successfully catheterized owing to difficult aortic arch anatomy. 1 C: Target occlusion was not reached due to the inability to pass a cervical ICA occlusion (e.g. unpassable tandem lesion).
2	**Technical reasons: Target occlusion reached** 2 A: Target occlusion was reached, but the operator was unable to pass the thrombus with the microwire/microcatheter. In these cases, no stent retriever was deployed. 2B: Target occlusion was reached, the stent retriever was deployed, but no reperfusion occurred after multiple retrievals (no clot retrieval or dislocation), thus, stent retriever failure. 2 C: Initial reperfusion was achieved, followed by spontaneous or iatrogenic reocclusion (e.g. intracranial stenosis, intracranial dissection, or perforation with subsequent vessel sacrifice).
3	**Non-technical reasons** Presumed futility. Adverse non-neurological event with the need to stop mechanical thrombectomy. Signs of contrast extravasation without perforation (early hemorrhagic transformation). *Puncture of a suitable vessel for access route not possible.* *Periinterventional arterial dissection.* *Classification is not feasible based on the available documents.*

Legend: ICA - internal carotid artery

Given the urgency of more detailed understanding of MT failure, our large retrospective single center study from a German university hospital aims to comprehensively analyze demographic characteristics (age, sex, pre- and post-MT modified Rankin Scale [mRS] values, bridging intravenous thrombolysis [IVT] with plasminogen activator) and vessel occlusion site (anterior vs. posterior circulation respectively proximal vs. distal segments) as well as other risk factors for MT failure according to the classification of Kaesmacher et al. In addition, we carried out an analysis of MT failure over time.

## Methods

### Study design

In a retrospective analysis we evaluated 552 patients in total who underwent MT due to AIS in the anterior or posterior cerebral circulation between August 2019 and March 2023 in our high-volume comprehensive stroke center of a German university hospital. Our standard MT approach was as follows: After induction of general anesthesia, a long 6 french (F) sheath is placed in the internal carotid artery (ICA) or vertebral artery via femoral access with a 5 F guide catheter. An appropriately sized aspiration catheter is placed at the face of the thrombus in a triaxial approach and two attempts of direct aspiration are performed. If recanalization was unsuccessful, the decision to employ an additional stent retrieval technique, repeat the aspiration maneuver or to end the interventional procedure was made by the neuro-interventionalist.

Mechanical thrombectomy failure was defined as mTICI score of 0 or 1.

Patients with MT failure were analyzed for demographic characteristics age, sex, mRS values at admission (pre-MT) and at discharge from hospital (post-MT), bridging IVT and site of vessel occlusion (anterior vs. posterior circulation, proximal ICA, M1, M2, A1, A2, basilar artery [BA], P1 vs. distal [A3, M3, P2] segments) as well as reasons for MT failure according to the classification of Kaesmacher et al. [15, 19]. In addition, we analyzed MT failure of patients over time.

All angiographic results were evaluated by two experienced neuro-interventionalists with board certification.

Approval of our institutional review board was obtained, whereby research ethics and informed consent were considered (file reference: 2023 − 662).

### Statistics

Statistical analyses were performed using R (version 4.3.1). Pearson’s Chi-square tests (parametric) were employed for categorical variables, logistic regression (parametric) for binary outcomes and Wilcoxon rank-sum and signed-rank tests (non-parametric) for ordinal data. Fisher’s exact test (non-parametric) was used for categorical data and ordinal logistic regression (parametric) for ordered categorical outcomes. The alpha level was set at 0.05, with p-values less than this threshold considered statistically significant.

## Results

### All vessel occlusions and mechanical thrombectomies

In all 552 patients receiving MT, 469 vessel occlusions (85%) occurred in the anterior circulation and 73 (13.2%) in the posterior circulation. The 10 remaining cases (1.8%) constituted combined occlusions in different territories, whereby two vessel segments alone were affected at the same time (each without simultaneous MT). The occlusion leading to thrombectomy was found in a proximal vessel segment in 527 cases (95.5%) and in a distal vessel segment in 25 cases (4.5%; A3 *n* = 1 [left], M3 *n* = 9 [8 left, 1 right], P2 *n* = 15 [9 left, 6 right]). 496/552 patients (89.9%) showed successful MT after use of direct aspiration and/or stent retriever, with *n* = 29 (5.8%) undergoing intracranial rescue stenting.

### Mechanical thrombectomy failure patients

#### Demographic characteristics and site of vessel occlusion (anterior vs. posterior circulation, proximal vs. distal segments)

Mechanical thrombectomy failure occurred in 56 patients (10.1%; *n* = 47 anterior circulation, *n* = 9 posterior circulation). In these cases, 49 patients (87.5%) finally showed mTICI 0, while in 7 patients (12.5%) MT resulted in mTICI 1. There was no case with rescue stenting that resulted in MT failure. The median age of the patients with an unsuccessful MT was 76 (interquartile range: 69.75-82). Thirty patients (53.6%) with MT failure were female and *n* = 26 male (46.4%). The distribution of mRS values at admission for patients with MT failure showed a median of 4 (range 1–5), whereas the median at discharge from hospital was 5 (range 2–6). Nineteen patients (33.9%) with MT failure received bridging IVT, while *n* = 37 (66.1%) did not. There were 48 cases of failed thrombectomies in proximal (85.7%) and 8 (14.3%) in distal vessel occlusions, with M2 branch being the most frequent location of MT failure. Site distribution of MT failure in detail is presented in Table 3.

**Table 3:** Site distribution of mechanical thrombectomy failure

Vessel occlusion	Left (*n*)	Right (*n*)
ICA	6	4
M1	7	4
M2	9	8
M3	3	0
A1	1	0
A2	1	3
A3	1	0
BA	1	
P1	3	2
P2	1	2

Legend: BA - basilar artery, ICA - internal carotid artery, mTICI - modified thrombolysis in cerebral infarction scale

Review of MT failure, according to the Kaesmacher classification, shows that stent retriever failure was the most frequent cause in our study cohort (category 2B: 27/56 patients [48%]). The detailed distribution is listed in Table 4.

**Table 4:**
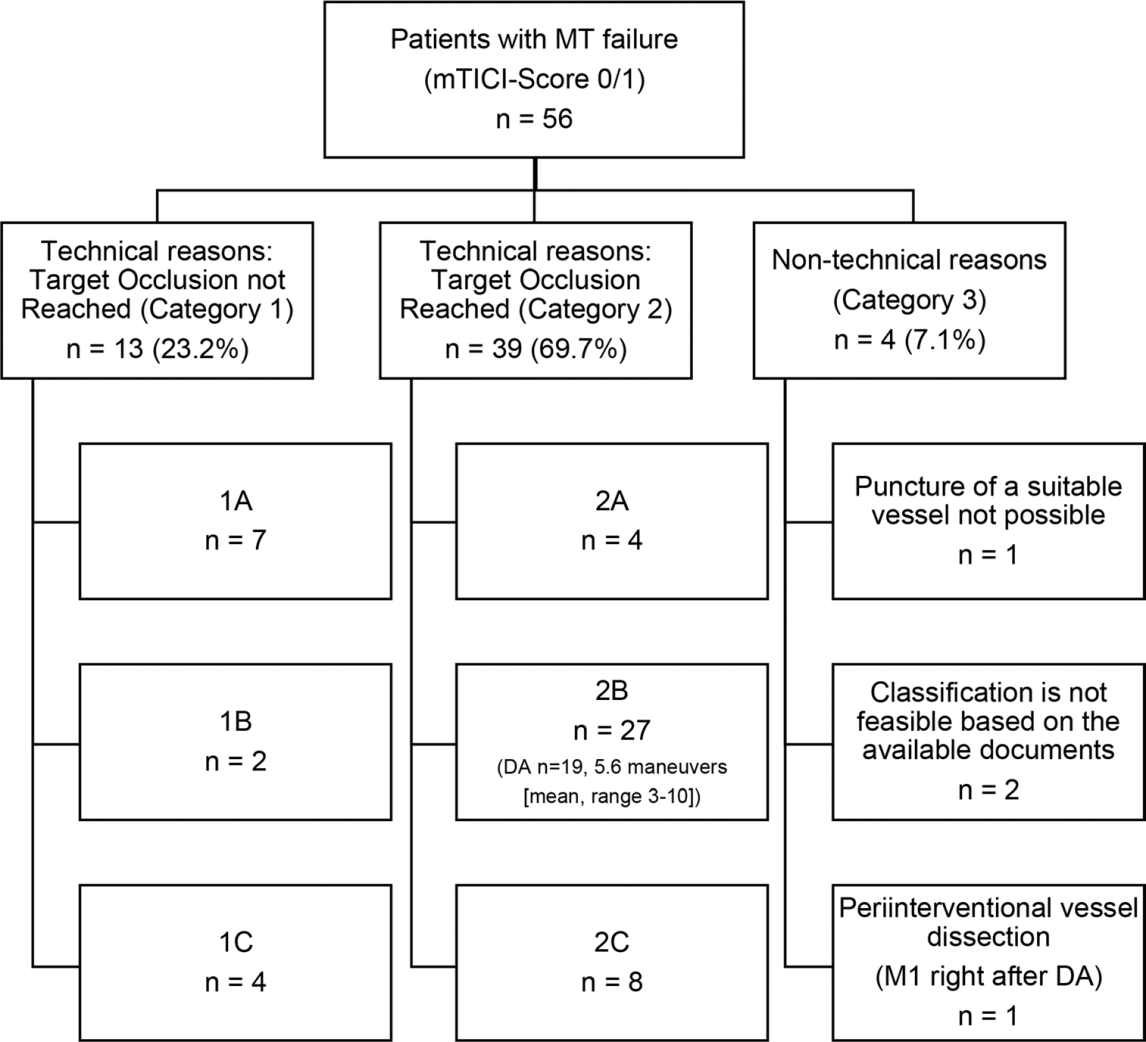
Mechanical thrombectomy failure reasons in our study using the classification system of Kaesmacher et al. as supplemented by us [19]

#### Specific statistical analyses including the classification of Kaesmacher et al. and trend over time

A logistic regression analysis showed a statistically non-significant trend towards a negative association of age with MT failure (*p* = 0.165). We found no significant association between sex and unsuccessful endovascular recanalization (*p* = 0.738). Bridging IVT was not significantly associated with successful MT (*p* = 0.326). An improvement in the clinical outcome on the basis of pre- and post-MT mRS was significantly associated with thrombectomy success (TICI ≥ 2a; *p* < 0.001). There was also no correlation of vessel occlusion localization in the anterior or posterior circulation on angiographic reperfusion after MT (*p* = 0.838). However, distal vessel occlusions (A3, M3, P2) generally demonstrated lower MT success rates than proximal occlusions (ICA, M1, M2, A1, A2, VA, BA, P1) (*p* < 0.01).

**Fig. 1:**
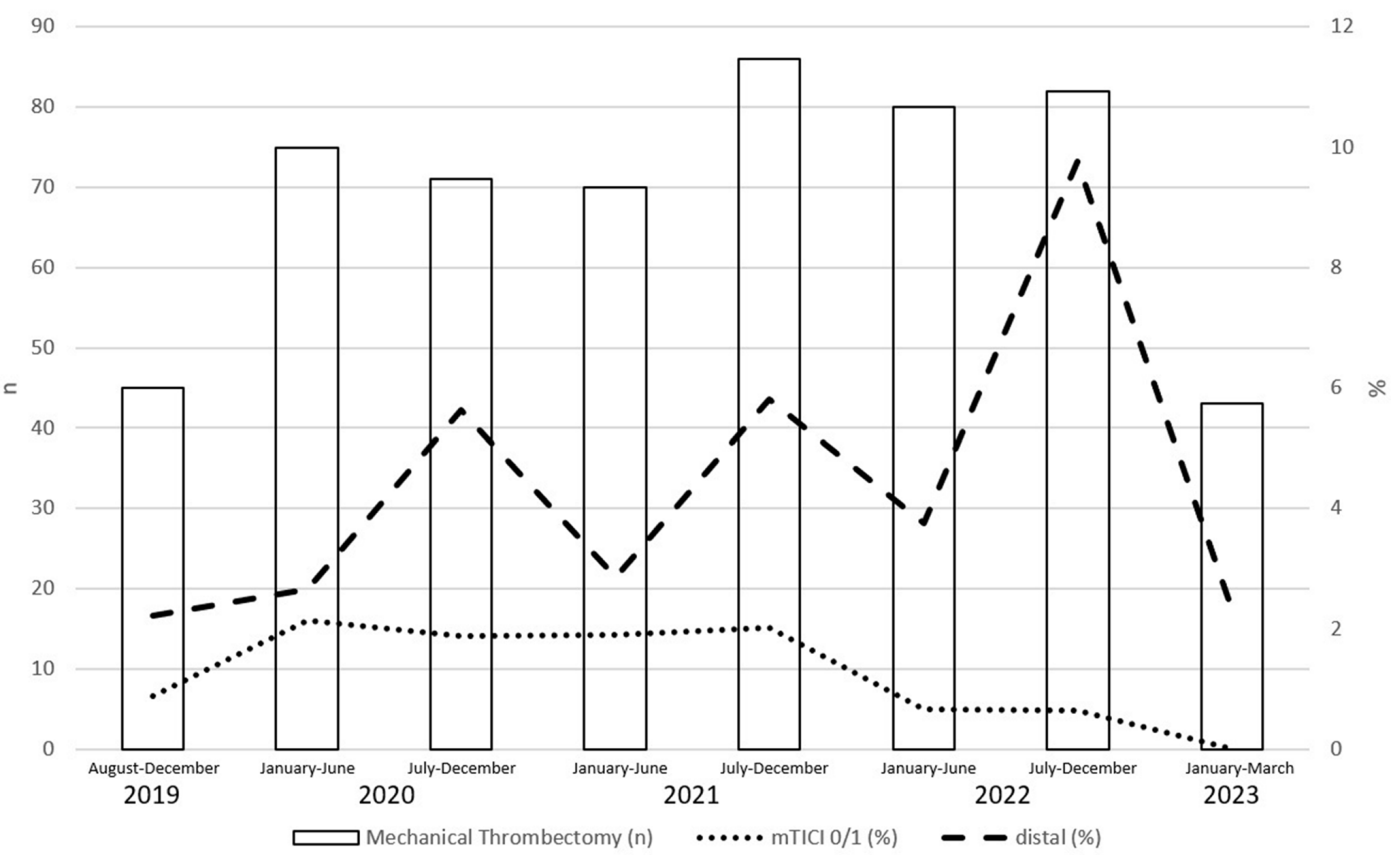
Mechanical thrombectomy failure over time in our study. *Legend*: MT - mechanical thrombectomy

Our review of MT failure in detail, according to the Kaesmacher classification, showed that stent retriever failure was the most frequent cause (category 2a: 48%, *p* < 0.001). Furthermore, demographic factors such as age or sex showed no correlation across different failure types (*p* = 0.610, *p* = 0.805). Concerning the success rates of MT in different calendar years, the results indicated significant differences between 2020 (MT failure *n* = 22) and 2022 (*n* = 8, *p* < 0.01), 2020 (*n* = 22) and 2023 (*n* = 0, *p* < 0.01), 2021 (*n* = 23) and 2022 (*n* = 8, *p* < 0.01) as well as 2021 (*n* = 23) and 2023 (*n* = 8, *p* < 0.01; Fig. 1). Comparisons between other years did not yield statistically significant findings (*p* > 0.05).

## Discussion

Our aim was to analyze patients with MT failure. Considering this ongoing and dynamic issue, we present results from a large single center study conducted in a high-volume comprehensive center of a German university hospital, including both anterior and posterior circulation strokes. Mechanical thrombectomy failure patients were analyzed for baseline characteristics (age, sex, clinical conditions, bridging IVT) as well as vessel occlusion localization (anterior vs. posterior circulation, proximal [ICA, M1, M2, A1, A2, VA, BA, P1] vs. distal [A3, M3, P2] segments) and other reasons for MT failure according to the classification of Kaesmacher et al. In addition, we carried out an analysis of MT failure over time.

Unsuccessful endovascular recanalization is a well-recognized challenge in the treatment of acute ischemic stroke, with prior studies reporting failure rates ranging from 10.6 to 26.1%, based on the mTICI score [18–26]. Overall, our MT success rate is in line with the results of other work groups, although MT failure was not consistently defined across studies (mTICI 0/1 vs. 0-2a), making comparisons difficult in some cases **(18**,** 19**,** 20–26;** Table 1**)**.

Our logistic regression analysis showed a trend towards a negative association of age with MT failure (*p* = 0.165) and we found no significant association between sex and unsuccessful endovascular recanalization (*p* = 0.738). Different from this finding, Goda et al. identified female sex as a predictor of MT failure [18]. Furthermore, in our results, bridging IVT was not significantly associated with MT success (*p* = 0.326), partly arguing against recent results from the SWIFT DIRECT trial that supported IVT additionally to MT for AIS treatment [31]. However, our data did not result from a randomized trial. Therefore, there is a clear selection bias between both groups that might influence results.

Our study further enhances understanding of MT results across different vascular territories. Several studies have already dealt with this; we refer to a meta-analysis of Adusumilli presenting comparable mechanical recanalization rates in anterior and posterior circulation [32]. Also, our results did not show a correlation of site of vessel occlusion in the different circulations on angiographic reperfusion after MT (*p* = 0.838). The lack of significant differences between the territories suggests that thrombus location does not compromise MT success, which is in a way in line with results of registry data [33].

We also compared the proximal and distal distribution in relation to thrombectomy success. In our study, the exploration of MT outcomes by the location of occlusion reveals that thrombectomy targeting distal vessels (in our cases A3, M3 and P2) have significantly lower recanalization rates compared to proximal thrombus locations (*p* < 0.01). In contrast, a systematic review indicates satisfactory MT outcomes for more distal cerebral arteries [34]. Nevertheless, the difference revealed in our study obviously confirms that distal occlusions of small vessels are more challenging, potentially due to increased tortuosity, resulting in less accessibility for MT devices, which may be more difficult to navigate, therefore increasing the complexity of the endovascular procedure. In the case of distal emboli, we see a higher number of left-brain findings in our results (18 of 25 cases). In this context, the literature predominantly describes more effective MT outcomes for LVO associated with left cerebral ischemia [35].

While previous work such as that by Heider et al. has already examined mechanisms and co-factors of MT failure in detail, we would also like to add new aspects to the topic [24]. The additional classification by Kaesmacher et al., which distinguishes between technical and non-technical reasons for MT failure, offers valuable insights into the factors contributing to MT outcomes [19]. We have also used this classification in our collective for patients treated with direct aspiration in connection with AIS. Our review of factors associated with unsuccessful thrombectomies according to the proposal of Kaesmacher et al. shows that stent retriever failure, despite correct positioning and potentially multiple retrieval attempts, is the primary cause (category 2B: 27/56 patients [48%]) and this demonstrated significant differences to the other categories (*p* < 0.001). Notably, our findings indicate a higher failure rate than reported by Kaesmacher et al., who noted stent retriever failure in 39.7% of their 63 patients [17]. This highlights the need for even greater emphasis on further development of stent retrievers, with a focus more on the specific thrombus optimized devices [30]. However, this kind of MT failure directly at the occlusion may be influenced by challenging vascular anatomy, such as tight curves or extensive atherosclerosis, which complicates the retrieval process itself [28, 30]. Furthermore, especially for the patients classified by us according to Kaesmacher et al., the demographic factors age and sex showed no correlation across different failure types (*p* = 0.610, *p* = 0.805). This suggests that they may influence overall stroke outcomes, but not directly impact MT success.

Lastly and as a further new aspect for literature, our time trend results suggest improving MT success rates in the further course. This is particularly noteworthy in the period from 2021 to 2023, with not a single failure documented in the last year in 43 MT patients examined (Fig. 4). It is worth mentioning that a relatively long period of time (almost 4 years) is included in this study, in which the development of new tools has likely played a role in decreasing the number of MT failures over time. The observed improvements in endovascular recanalization rates may be attributed in part to the increased use of thrombectomy especially for distal vessel occlusions with enhanced knowledge of material handling. As neuro-interventionalists become more familiar with the techniques of navigating and using these technologically advanced devices, they are able to perform MT more successfully and with better outcomes [36]. Especially for distal occlusions, therefore, advancements in devices, particularly stent retrievers, are crucial in shaping the future of MT [37]. Temporal trends in thrombectomy success have so far only been systematically investigated for LVO [38].

In general, additive MT procedures through other access routes (radial or direct ICA puncture) present a promising approach as an alternative strategy in cases of initial procedural failure via femoral artery access and certainly require further investigation [39–41]. Additionally, the utilization of simulators capable of replicating intricate anatomical scenarios could provide valuable training opportunities [42]. Furthermore, recent studies have shown that the use of intra-arterial tissue-type plasminogen activators after unsuccessful MT did not result in a higher risk of symptomatic intracranial hemorrhage or mortality, making it a low-risk bailout procedure [43, 44].

## Limitations

This study’s retrospective design with single-center focus, different vessel occlusion durations especially due to “drip- and-ship”, use of varying procedural techniques for MT with changing experience levels of the neuro-interventionalists (learning curves) and lack of longer-term follow-up data limits its generalizability and introduces potential biases. As retrospective studies carry inherent limitations, more prospective studies on this topic are crucial.

The mTICI score was not originally intended for the description of therapy results of the posterior circulation, but is also widely used for this purpose [16]. In addition, there are different definitions for distal vessel occlusions and also overlaps with Medium Vessel Occlusions (MeVO), and we have referred to the descriptions of the multicenter initiative Stroke Thrombectomy and Aneurysm Registry (STAR) [15, 45, 46]. In the retrospective approach applied here, the exact reason for a persistent vessel occlusion after MT and possibly the lack of a change in interventional strategy with consideration of rescue stenting, among others, could not be clearly traced, although there are also novel developments in this area in particular [47–50]. Furthermore, in future the goal should be to better assess the additive effect of bridging IVT depending on localization of the vessel occlusion to be mechanically recanalized and achieve a better understanding of predictors for MT failure, particularly in very old patients [51, 52].

Beyond that, with regard to outcome, predictive clinical and radiological parameters [53] as well as other influences such as anticoagulation [54], tandem occlusions with simultaneous emergent ICA stenting [55], collaterals [56] and secondary intracerebral hemorrhages [57, 58] have not been analyzed by us. However, these will certainly also be the subject of future research. In addition, the question of whether the duration of the interventional procedure and the number of thrombectomy maneuvers have a decisive influence on the outcome in the case of unsuccessful endovascular recanalization was also discussed very recently, which was not considered in our survey [59].

## Data Availability

The data that support the findings of this study are available from the authors Alexander Neumann and Paul Schildhauer, upon reasonable request.

## References

[1] Munoz A, Jabre R, Orenday-Barraza JM, Eldin MS, Chen CJ, Al-Saiegh F, Abbas R, El Naamani K, Gooch MR, Jabbour PM, Tjoumakaris S, Rosenwasser RH, Herial NA (2023) A review of mechanical thrombectomy techniques for acute ischemic stroke. Interv Neuroradiol 29(4):450–458 Epub 2022 Mar 3. PMID: 35238227; PMCID: PMC10399505 35238227 10.1177/15910199221084481 PMC10399505

[2] Tao C, Nogueira RG, Zhu Y, Sun J, Han H, Yuan G, Wen C, Zhou P, Chen W, Zeng G, Li Y, Ma Z, Yu C, Su J, Zhou Z, Chen Z, Liao G, Sun Y, Ren Y, Zhang H, Chen J, Yue X, Xiao G, Wang L, Liu R, Liu W, Liu Y, Wang L, Zhang C, Liu T, Song J, Li R, Xu P, Yin Y, Wang G, Baxter B, Qureshi AI, Liu X, Hu W (2022) ATTENTION Investigators. Trial of Endovascular Treatment of Acute Basilar-Artery Occlusion. N Engl J Med.;387(15):1361–1372. 10.1056/NEJMoa2206317. PMID: 36239644 10.1056/NEJMoa2206317 36239644

[3] Zhang Y, Zhang Y, Hu C, Zhao W, Zhang Z, Li W (2021) A direct aspiration first-pass technique (ADAPT) versus stent retriever for acute ischemic stroke (AIS): a systematic review and meta-analysis. J Neurol 268(12):4594–4606. 10.1007/s00415-020-10284-wEpub 2020 Oct 29. PMID: 33123777 33123777 10.1007/s00415-020-10284-w

[4] Roaldsen MB, Jusufovic M, Berge E, Lindekleiv H (2021) Endovascular thrombectomy and intra-arterial interventions for acute ischaemic stroke. Cochrane Database Syst Rev 6(6):CD007574. 10.1002/14651858.CD007574.pub3PMID: 34125952; PMCID: PMC8203212 34125952 10.1002/14651858.CD007574.pub3 PMC8203212

[5] Powers WJ, Rabinstein AA, Ackerson T, Adeoye OM, Bambakidis NC, Becker K, Biller J, Brown M, Demaerschalk BM, Hoh B, Jauch EC, Kidwell CS, Leslie-Mazwi TM, Ovbiagele B, Scott PA, Sheth KN, Southerland AM, Summers DV, Tirschwell DL (2019) Guidelines for the Early Management of Patients With Acute Ischemic Stroke: 2019 Update to the 2018 Guidelines for the Early Management of Acute Ischemic Stroke: A Guideline for Healthcare Professionals From the American Heart Association/American Stroke Association. Stroke.;50(12):e344-e418. doi: 10.1161/STR.0000000000000211. Epub 2019 Oct 30. Erratum in: Stroke. 2019;50(12):e440-e441. PMID: 31662037

[6] Turc G, Bhogal P, Fischer U, Khatri P, Lobotesis K, Mazighi M, Schellinger PD, Toni D, de Vries J, White P, Fiehler J (2019) European Stroke Organisation (ESO) - European Society for Minimally Invasive Neurological Therapy (ESMINT) guidelines on mechanical thrombectomy in Acute Ischaemic StrokeEndorsed by Stroke Alliance for Europe (SAFE). Eur Stroke J 4(1):6–12 Epub 2019 Feb 26. PMID: 31165090; PMCID: PMC6533858 31165090 10.1177/2396987319832140 PMC6533858

[7] Costalat V, Jovin TG, Albucher JF, Cognard C, Henon H, Nouri N, Gory B, Richard S, Marnat G, Sibon I, Di Maria F, Annan M, Boulouis G, Cardona P, Obadia M, Piotin M, Bourcier R, Guillon B, Godard S, Pasco-Papon A, Eker OF, Cho TH, Turc G, Naggara O, Velasco S, Lamy M, Clarençon F, Alamowitch S, Renu A, Suissa L, Brunel H, Gentric JC, Timsit S, Lamy C, Chivot C, Macian-Montoro F, Mounayer C, Ozkul-Wermester O, Papagiannaki C, Wolff V, Pop R, Ferrier A, Chabert E, Ricolfi F, Béjot Y, Lopez-Cancio E, Vega P, Spelle L, Denier C, Millán M, Arenillas JF, Mazighi M, Houdart E (2024) Mar Freijo M, Duhamel A, Sanossian N, Liebeskind DS, Labreuche J, Lapergue B, Arquizan C; LASTE Trial Investigators. Trial of Thrombectomy for Stroke with a Large Infarct of Unrestricted Size. N Engl J Med.;390(18):1677–1689. 10.1056/NEJMoa2314063. PMID: 38718358 10.1056/NEJMoa2314063 38718358

[8] Winkelmeier L, Maros M, Flottmann F, Heitkamp C, Schön G, Thomalla G, Fiehler J, Hanning U (2024) Endovascular Thrombectomy for Large Ischemic Strokes with ASPECTS 0–2: a Meta-analysis of Randomized Controlled Trials. Clin Neuroradiol. Apr 30. 10.1007/s00062-024-01414-2. Epub ahead of print. PMID: 38687364 10.1007/s00062-024-01414-2 PMC11339095 38687364

[9] Khunte M, Chen H, Khunte A, Payabvash S, Gandhi D, Malhotra A (2023) Trends in Use of Intravenous Thrombolysis and Endovascular Thrombectomy in patients with Acute Stroke with large vessel occlusion 2016 to 2020 and impact of COVID-19 pandemic. J Am Heart Assoc 12(21):e029579 Epub 2023 Oct 27. PMID: 37889182; PMCID: PMC10727381 37889182 10.1161/JAHA.122.029579 PMC10727381

[10] Rodriguez-Calienes A, Galecio-Castillo M, Vivanco-Suarez J, Mohamed GA, Toth G, Sarraj A, Pujara D, Chowdhury AA, Farooqui M, Ghannam M, Samaniego EA, Jovin TG, Ortega-Gutierrez S Endovascular thrombectomy beyond 24 hours from last known well: a systematic review with meta-analysis. J Neurointerv Surg 2023 Jun 24:jnis–2023. 10.1136/jnis-2023-020443. Epub ahead of print. PMID: 37355251. 10.1136/jnis-2023-020443 37355251

[11] Li L, Scott CA, Rothwell PM (2020) Oxford Vascular Study. Trends in Stroke incidence in high-income countries in the 21st Century: Population-based study and systematic review. Stroke 51(5):1372–1380. 10.1161/STROKEAHA.119.028484Epub 2020 Mar 25. PMID: 32208842; PMCID: PMC7185053 32208842 10.1161/STROKEAHA.119.028484 PMC7185053

[12] Bai X, Zhang X, Zhang Y, Yang W, Wang T, Feng Y, Wang Y, Yang K, Wang X, Ma Y, Jiao L (2021) Mechanical thrombectomy in nonagenarians: a systematic review and Meta-analysis. Transl Stroke Res 12(3):394–405 Epub 2021 Feb 2. PMID: 33532934 33532934 10.1007/s12975-021-00894-5

[13] Sporns PB, Sträter R, Minnerup J, Wiendl H, Hanning U, Chapot R, Henkes H, Henkes E, Grams A, Dorn F, Nikoubashman O, Wiesmann M, Bier G, Weber A, Broocks G, Fiehler J, Brehm A, Psychogios M, Kaiser D, Yilmaz U, Morotti A, Marik W, Nolz R, Jensen-Kondering U, Schmitz B, Schob S, Beuing O, Götz F, Trenkler J, Turowski B, Möhlenbruch M, Wendl C, Schramm P, Musolino P, Lee S, Schlamann M, Radbruch A, Rübsamen N, Karch A, Heindel W, Wildgruber M, Kemmling A (2020) Feasibility, Safety, and Outcome of Endovascular recanalization in Childhood Stroke: the Save ChildS Study. JAMA Neurol 77(1):25–34. 10.1001/jamaneurol.2019.3403PMID: 31609380; PMCID: PMC6802048 31609380 10.1001/jamaneurol.2019.3403 PMC6802048

[14] Clarençon F, Durand-Zaleski I, Premat K, Baptiste A, Chabert E, Ferrier A, Labeyrie MA, Reiner P, Spelle L, Denier C, Tuilier T, Hosseini H, Rodriguez-Régent C, Turc G, Fauché C, Lamy M, Lapergue B, Consoli A, Barbier C, Boulanger M, Bricout N, Henon H, Gory B, Richard S, Rouchaud A, Macian-Montoro F, Eker O, Cho TH, Soize S, Moulin S, Gentric JC, Timsit S, Darcourt J, Albucher JF, Janot K, Annan M, Pico F, Costalat V, Arquizan C, Marnat G, Sibon I, Pop R, Wolff V, Shotar E, Lenck S, Sourour NA, Radenne A, Alamowitch S, Dechartres A (2024) Evaluation of mechanical thrombectomy in acute ischemic stroke related to a distal arterial occlusion: a randomized controlled trial. Int J Stroke 19(3):367–372. 10.1177/17474930231205213Epub 2023 Oct 12. PMID: 37740419 37740419 10.1177/17474930231205213

[15] Alawieh AM, Chalhoub RM, Al Kasab S, Jabbour P, Psychogios MN, Starke RM, Arthur AS, Fargen KM, De Leacy R, Kan P, Dumont TM, Rai A, Crosa RJ, Maier I, Goyal N, Wolfe SQ, Cawley CM, Mocco J, Tjoumakaris SI, Howard BM, Dimisko L, Saad H, Ogilvy CS, Crowley RW, Mascitelli JR, Fragata I, Levitt MR, Kim JT, Park MS, Gory B, Polifka AJ, Matouk C, Grossberg JA, Spiotta AM (2023) STAR Collaborators. Multicenter investigation of technical and clinical outcomes after thrombectomy for distal vessel occlusion by frontline technique. J Neurointerv Surg 15(e1):e93–e101. 10.1136/jnis-2022-019023Epub 2022 Aug 2. PMID: 35918129 35918129 10.1136/jnis-2022-019023

[16] Meyer L, Stracke CP, Jungi N, Wallocha M, Broocks G, Sporns PB, Maegerlein C, Dorn F, Zimmermann H, Naziri W, Abdullayev N, Kabbasch C, Behme D, Jamous A, Maus V, Fischer S, Möhlenbruch M, Weyland CS, Langner S, Meila D, Miszczuk M, Siebert E, Lowens S, Krause LU, Yeo LLL, Tan BY, Anil G, Gory B, Galván J, Arteaga MS, Navia P, Raz E, Shapiro M, Arnberg F, Zelenák K, Martinez-Galdamez M, Fischer U, Kastrup A, Roth C, Papanagiotou P, Kemmling A, Gralla J, Psychogios MN, Andersson T, Chapot R, Fiehler J, Kaesmacher J, Hanning U (2021) Thrombectomy for primary distal posterior cerebral artery occlusion stroke: the TOPMOST study. JAMA Neurol 78(4):434–444. 10.1001/jamaneurol.2021.0001PMID: 33616642; PMCID: PMC7900924 33616642 10.1001/jamaneurol.2021.0001 PMC7900924

[17] Saver JL, Chapot R, Agid R, Hassan A, Jadhav AP, Liebeskind DS, Lobotesis K, Meila D, Meyer L, Raphaeli G, Gupta R (2020) Distal Thrombectomy Summit Group*†. Thrombectomy for Distal, Medium Vessel Occlusions: A Consensus Statement on Present Knowledge and Promising Directions. Stroke.;51(9):2872–2884. 10.1161/STROKEAHA.120.028956. Epub 2020 Aug 6. Erratum in: Stroke. 2020;51(10):e296. PMID: 32757757 10.1161/STROKEAHA.120.028956 32757757

[18] Goda T, Oyama N, Kitano T, Iwamoto T, Yamashita S, Takai H, Matsubara S, Uno M, Yagita Y (2019) Factors Associated with unsuccessful recanalization in mechanical thrombectomy for Acute ischemic stroke. Cerebrovasc Dis Extra 9(3):107–113 Epub 2019 Sep 27. PMID: 31563915; PMCID: PMC6792430 31563915 10.1159/000503001 PMC6792430

[19] Kaesmacher J, Gralla J, Mosimann PJ, Zibold F, Heldner MR, Piechowiak E, Dobrocky T, Arnold M, Fischer U, Mordasini P (2018) Reasons for reperfusion failures in Stent-Retriever-based thrombectomy: Registry analysis and proposal of a classification system. AJNR Am J Neuroradiol 39(10):1848–1853. 10.3174/ajnr.A5759Epub 2018 Aug 30. PMID: 30166434; PMCID: PMC7410746 30166434 10.3174/ajnr.A5759 PMC7410746

[20] Zaidat OO, Yoo AJ, Khatri P, Tomsick TA, von Kummer R, Saver JL, Marks MP, Prabhakaran S, Kallmes DF, Fitzsimmons BF, Mocco J, Wardlaw JM, Barnwell SL, Jovin TG, Linfante I, Siddiqui AH, Alexander MJ, Hirsch JA, Wintermark M, Albers G, Woo HH, Heck DV, Lev M, Aviv R, Hacke W, Warach S, Broderick J, Derdeyn CP, Furlan A, Nogueira RG, Yavagal DR, Goyal M, Demchuk AM, Bendszus M, Liebeskind DS; Cerebral Angiographic Revascularization Grading (CARG) Collaborators; STIR Revascularization working group; STIR Thrombolysis in Cerebral Infarction (TICI) Task Force. Recommendations on angiographic revascularization grading standards for acute ischemic stroke: a consensus statement. Stroke.;44(9):2650-63. doi:, Almallouhi O, Ali E, Essibayi H, Bass MA, Neyens E, Anadani R, Chalhoub M, Kicielinski R, Lena K, Porto J, Sattur G, Spiotta M, Kasab AM (2013) SA. Failed mechanical thrombectomy: prevalence, etiology, and predictors. J Neurosurg. 2023;139(3):714–720. doi: 10.3171/2022.12.JNS222152. PMID: 36670537 10.3171/2022.12.JNS222152 36670537

[21] Marnat G, Gory B, Sibon I, Kyheng M, Labreuche J, Boulouis G, Liegey JS, Caroff J, Eugène F, Naggara O, Consoli A, Mazighi M, Maier B, Richard S, Denier C, Turc G, Lapergue B, Bourcier R (2022) Endovascular treatment in ischemic stroke (ETIS) investigators. Mechanical thrombectomy failure in anterior circulation strokes: outcomes and predictors of favorable outcome. Eur J Neurol 29(9):2701–2707. 10.1111/ene.15429Epub 2022 Jun 18. PMID: 35648445; PMCID: PMC9541042 35648445 10.1111/ene.15429 PMC9541042

[22] Flottmann F, Broocks G, Faizy TD, McDonough R, Watermann L, Deb-Chatterji M, Thomalla G, Herzberg M, Nolte CH, Fiehler J, Leischner H, Brekenfeld C (2021) GSR investigators. Factors Associated with failure of reperfusion in endovascular therapy for Acute ischemic stroke: a Multicenter Analysis. Clin Neuroradiol 31(1):197–205 Epub 2020 Feb 17. PMID: 32067055; PMCID: PMC7943507 32067055 10.1007/s00062-020-00880-8 PMC7943507

[23] Weyland CS, Neuberger U, Potreck A, Pfaff JAR, Nagel S, Schönenberger S, Bendszus M, Möhlenbruch MA (2021) Reasons for failed mechanical thrombectomy in posterior circulation ischemic stroke patients. Clin Neuroradiol 31(3):745–752. 10.1007/s00062-020-00950-xEpub 2020 Sep 7. PMID: 32894352; PMCID: PMC8463404 32894352 10.1007/s00062-020-00950-x PMC8463404

[24] Heider DM, Simgen A, Wagenpfeil G, Dietrich P, Yilmaz U, Mühl-Benninghaus R, Roumia S, Faßbender K, Reith W, Kettner M (2020) Why we fail: mechanisms and co-factors of unsuccessful thrombectomy in acute ischemic stroke. Neurol Sci 41(6):1547–1555 Epub 2020 Jan 23. PMID: 31974796; PMCID: PMC7275938 31974796 10.1007/s10072-020-04244-5 PMC7275938

[25] Leischner H, Flottmann F, Hanning U, Broocks G, Faizy TD, Deb-Chatterji M, Bernhardt M, Brekenfeld C, Buhk JH, Gellissen S, Thomalla G, Gerloff C, Fiehler J (2019) Reasons for failed endovascular recanalization attempts in stroke patients. J Neurointerv Surg 11(5):439–442. 10.1136/neurintsurg-2018-014060Epub 2018 Nov 24. PMID: 30472671 30472671 10.1136/neurintsurg-2018-014060

[26] Penide J, Mirza M, McCarthy R, Fiehler J, Mordasini P, Delassus P, Morris L, Gilvarry M (2022) Systematic review on Endovascular Access to intracranial arteries for mechanical thrombectomy in Acute ischemic stroke. Clin Neuroradiol 32(1):5–12. 10.1007/s00062-021-01100-7Epub 2021 Oct 12. PMID: 34642788 34642788 10.1007/s00062-021-01100-7

[27] Gascou G, Lobotesis K, Machi P, Maldonado I, Vendrell JF, Riquelme C, Eker O, Mercier G, Mourand I, Arquizan C, Bonafé A, Costalat V (2014) Stent retrievers in acute ischemic stroke: complications and failures during the perioperative period. AJNR Am J Neuroradiol 35(4):734–740. 10.3174/ajnr.A3746Epub 2013 Oct 24. PMID: 24157734; PMCID: PMC7965801 24157734 10.3174/ajnr.A3746 PMC7965801

[28] Alverne FJAM, Lima FO, Rocha FA, Bandeira DA, Lucena AF, Silva HC, Lee JS, Nogueira RG (2020) Unfavorable vascular anatomy during Endovascular Treatment of Stroke: challenges and Bailout strategies. J Stroke 22(2):185–202. 10.5853/jos.2020.00227Epub 2020 May 31. PMID: 32635684; PMCID: PMC7341011 32635684 10.5853/jos.2020.00227 PMC7341011

[29] Madjidyar J, Pineda Vidal L, Larsen N, Jansen O (2020) Influence of Thrombus composition on Thrombectomy: ADAPT vs. Balloon Guide Catheter and Stent Retriever in a Flow Model. Rofo 192(3):257–263 English. 10.1055/a-0998-4246 31514211 10.1055/a-0998-4246

[30] Kim BM (2017) Causes and solutions of Endovascular Treatment failure. J Stroke 19(2):131–142. 10.5853/jos.2017.00283Epub 2017 May 31. PMID: 28592777; PMCID: PMC5466284 28592777 10.5853/jos.2017.00283 PMC5466284

[31] Fischer U, Kaesmacher J, Strbian D, Eker O, Cognard C, Plattner PS, Bütikofer L, Mordasini P, Deppeler S, Pereira VM, Albucher JF, Darcourt J, Bourcier R, Benoit G, Papagiannaki C, Ozkul-Wermester O, Sibolt G, Tiainen M, Gory B, Richard S, Liman J, Ernst MS, Boulanger M, Barbier C, Mechtouff L, Zhang L, Marnat G, Sibon I, Nikoubashman O, Reich A, Consoli A, Lapergue B, Ribo M, Tomasello A, Saleme S, Macian F, Moulin S, Pagano P, Saliou G, Carrera E, Janot K, Hernández-Pérez M, Pop R, Schiava LD, Luft AR, Piotin M, Gentric JC, Pikula A, Pfeilschifter W, Arnold M, Siddiqui AH, Froehler MT, Furlan AJ, Chapot R, Wiesmann M, Machi P, Diener HC, Kulcsar Z, Bonati LH, Bassetti CL, Mazighi M, Liebeskind DS, Saver JL, Gralla J (2022) SWIFT DIRECT Collaborators. Thrombectomy alone versus intravenous alteplase plus thrombectomy in patients with stroke: an open-label, blinded-outcome, randomised non-inferiority trial. Lancet.;400(10346):104–115. 10.1016/S0140-6736(22)00537-2. PMID: 35810756 10.1016/S0140-6736(22)00537-2 35810756

[32] Adusumilli G, Pederson JM, Hardy N, Kallmes KM, Hutchison K, Kobeissi H, Heiferman DM, Heit JJ Mechanical thrombectomy in anterior vs. posterior circulation stroke: A systematic review and meta-analysis. Interv Neuroradiol. 2022 May 13:15910199221100796. doi: 10.1177/15910199221100796. Epub ahead of print. PMID: 35549748 10.1177/15910199221100796 PMC11310733 35549748

[33] Feil K, Berndt MT, Wunderlich S, Maegerlein C, Bernkopf K, Zimmermann H, Herzberg M, Tiedt S, Küpper C, Wischmann J, Schönecker S, Dimitriadis K, Liebig T, Dieterich M, Zimmer C, Kellert L, Boeckh-Behrens T (2023) GSR investigators. Endovascular thrombectomy for basilar artery occlusion stroke: analysis of the German Stroke Registry-Endovascular treatment. Eur J Neurol 30(5):1293–1302. 10.1111/ene.15694Epub 2023 Feb 22. PMID: 36692229 36692229 10.1111/ene.15694

[34] Bilgin C, Hardy N, Hutchison K, Pederson JM, Mebane A, Olaniran P, Kobeissi H, Kallmes KM, Fiorella D, Kallmes DF, Brinjikji W (2023) First-line thrombectomy strategy for distal and medium vessel occlusions: a systematic review. J Neurointerv Surg 15(6):539–546. 10.1136/jnis-2022-019344Epub 2022 Oct 12. PMID: 36223996 36223996 10.1136/jnis-2022-019344

[35] Li Z, Chu Z, Zhao S, Ma L, Yang Q, Huang X, Zhou Z (2019) Severe stroke patients with left-sided occlusion of the proximal anterior circulation benefit more from Thrombectomy. Front Neurol 10:551. 10.3389/fneur.2019.00551PMID: 31191440; PMCID: PMC6546891 31191440 10.3389/fneur.2019.00551 PMC6546891

[36] Hulscher F, Farouki Y, Mine B, Bonnet T, Wang M, Elens S, Suarez JV, Jodaitis L, Ligot N, Naeije G, Lubicz B, Guenego A (2022) Predictors of good clinical outcome after Thrombectomy for Distal Medium Vessel occlusions. World Neurosurg 160:e566–e572 Epub 2022 Jan 22. PMID: 35077884 35077884 10.1016/j.wneu.2022.01.067

[37] Guenego A, Mine B, Bonnet T, Elens S, Vazquez Suarez J, Jodaitis L, Ligot N, Naeije G, Lubicz B (2022) Thrombectomy for distal medium vessel occlusion with a new generation of Stentretriever (Tigertriever 13). Interv Neuroradiol 28(4):444–454 Epub 2021 Sep 13. PMID: 34516332; PMCID: PMC9326865 34516332 10.1177/15910199211039926 PMC9326865

[38] Bourcier R, Consoli A, Desilles JP, Labreuche J, Kyheng M, Desal H, Alias Q, Gory B, Dargazanli C, Janot K, Zhu F, Lapergue B, Marnat G (2023) Temporal trends in results of endovascular treatment of anterior intracranial large cerebral vessel occlusion: a 7-year study. Eur Stroke J 8(3):655–666 Epub 2023 Jun 8. PMID: 37288701; PMCID: PMC10472952 37288701 10.1177/23969873231180338 PMC10472952

[39] Collette SL, van de Ven EA, Luijckx GR, Lingsma HF, van Doormaal PJ, van Es ACGM, van den Wijngaard IR, Goldhoorn RB, de Groot JC, van Zwam WH, Majoie CBLM, Dippel DWJ, Bokkers RPH, Uyttenboogaart M, On Behalf Of The Mr Clean Registry Investigators (2023) Alternative arterial Access routes for Endovascular Thrombectomy in patients with Acute ischemic stroke: a study from the MR CLEAN Registry. J Clin Med 12(9):3257. 10.3390/jcm12093257PMID: 37176697; PMCID: PMC10179212 37176697 10.3390/jcm12093257 PMC10179212

[40] Hernandez D, Requena M, Olivé-Gadea M, de Dios M, Gramegna LL, Muchada M, García-Tornel Á, Diana F, Rizzo F, Rivera E, Rubiera M, Piñana C, Rodrigo-Gisbert M, Rodríguez-Luna D, Pagola J, Carmona T, Juega J, Rodríguez-Villatoro N, Molina C, Ribo M, Tomasello A (2024) Radial versus femoral Access for Mechanical Thrombectomy in patients with stroke: a Noninferiority Randomized Clinical Trial. Stroke 55(4):840–848 Epub 2024 Feb 1. PMID: 38527149 38299334 10.1161/STROKEAHA.124.046360

[41] Diel NJ, Gerner ST, Alhaj Omar O, Kalder J, Manz E, Keschenau PR, Struffert T, Brueckner T, Huttner HB, Doeppner TR (2023) Rendezvous intervention using combined surgical carotid endarterectomy followed by endovascular thrombectomy in patients with acute tandem occlusions: a proof-of-concept experience at a tertiary care center. Neurol Res Pract 5(1):60. 10.1186/s42466-023-00290-4PMID: 38057910; PMCID: PMC10701999 38057910 10.1186/s42466-023-00290-4 PMC10701999

[42] Paech D, Lehnen N, Lakghomi A, Schievelkamp A, Gronemann C, Bode FJ, Radbruch A, Dorn F (2023) School of Thrombectomy-A 3-Step Approach to perform Acute Stroke Treatment with Simulator training and virtual Supervision by Remote streaming support (RESS). Clin Neuroradiol 33(2):529–535 Epub 2022 Dec 15. PMID: 36520188; PMCID: PMC9753868 36520188 10.1007/s00062-022-01242-2 PMC9753868

[43] Zaidi SF, Castonguay AC, Zaidat OO, Mueller-Kronast N, Liebeskind DS, Salahuddin H, Jumaa MA (2021) Intra-arterial thrombolysis after unsuccessful mechanical thrombectomy in the STRATIS Registry. AJNR Am J Neuroradiol 42(4):708–712. 10.3174/ajnr.A6962Epub 2021 Jan 28. PMID: 33509921; PMCID: PMC8040984 33509921 10.3174/ajnr.A6962 PMC8040984

[44] Diprose WK, Wang MTM, Ghate K, Brew S, Caldwell JR, McGuinness B, Barber PA (2021) Adjunctive Intra-arterial Thrombolysis in Endovascular Thrombectomy: A Systematic Review and Meta-analysis. Neurology.;96(24):1135–1143. 10.1212/WNL.0000000000012112. PMID: 33931539 10.1212/WNL.0000000000012112 33931539

[45] Liu M, Nasr D, Brinjikji W (2023) The incidence of medium vessel occlusions: a population-based study. Front Neurol 14:1225066. 10.3389/fneur.2023.1225066PMID: 37576020; PMCID: PMC10415218 37576020 10.3389/fneur.2023.1225066 PMC10415218

[46] Ospel JM, Goyal M (2021) A review of endovascular treatment for medium vessel occlusion stroke. J Neurointerv Surg 13(7):623–630. 10.1136/neurintsurg-2021-017321Epub 2021 Feb 26. PMID: 33637570 33637570 10.1136/neurintsurg-2021-017321

[47] Rodriguez-Calienes A, Siddiqui FM, Galecio-Castillo M, Mohammaden MH, Dolia JN, Grossberg JA, Pabaney A, Hassan AE, Tekle WG, Saei H, Miller S, Majidi S, Fifi T, Valestin J, Siegler G, Penckofer JE, Zhang M, Sheth L, Salazar-Marioni SA, Iyyangar S, Nguyen A, Abdalkader TN, Linfante M, Dabus I, Mehta G, Sessa BP, Jumma J, Sugg MA, Linares RM, Nogueira G, Liebeskind RG, Haussen DS, Ortega-Gutierrez DC (2024 May) Rescue therapy for failed mechanical thrombectomy in Acute ischemic stroke: a pooled analysis of the Society of Vascular and Interventional Neurology Registry. Ann Neurol 16. 10.1002/ana.26967Epub ahead of print. PMID: 38752428 10.1002/ana.26967 38752428

[48] Martins PN, Nogueira RG, Tarek MA, Dolia JN, Sheth SA, Ortega-Gutierrez S, Salazar-Marioni S, Iyyangar A, Galecio-Castillo M, Rodriguez-Calienes A, Pabaney A, Grossberg JA, Haussen DC Early technique switch following failed passes during mechanical thrombectomy for ischemic stroke: should the approach change and when? J Neurointerv Surg. 2024 Apr 4:jnis-2024-021545. 10.1136/jnis-2024-021545. Epub ahead of print. PMID: 38479798. 10.1136/jnis-2024-021545 38479798

[49] Cai J, Xu H, Xiao R, Hu L, Xu P, Guo X, Xie Y, Pan M, Tang J, Gong Q, Liu Y, Su R, Deng J, Wang L (2023) Rescue intracranial stenting for acute ischemic stroke after the failure of mechanical thrombectomy: a systematic review, meta-analysis, and trial sequential analysis. Front Neurol 14:1023089. 10.3389/fneur.2023.1023089PMID: 36761342; PMCID: PMC9905111 36761342 10.3389/fneur.2023.1023089 PMC9905111

[50] Mosimann PJ, Kaesmacher J, Gautschi D, Bellwald S, Panos L, Piechowiak E, Dobrocky T, Zibold F, Mordasini P, El-Koussy M, Wiest R, Bervini D, Wagner F, Arnold M, Jung S, Galimanis A, Gralla A, Fischer U (2018) Predictors of unexpected early reocclusion after successful mechanical thrombectomy in Acute ischemic stroke patients. Stroke 49(11):2643–2651. 10.1161/STROKEAHA.118.021685. Erratum in: Stroke. 2018;49(12):e343. PMID: 30355192 10.1161/STROKEAHA.118.021685 30355192

[51] D’Anna L, Merlino G, Romoli M, Zhang L, Del Regno C, Aggour M, Levee V, Foschi M, Sponza M, Toraldo F, Algazlan R, Ruggiero M, Longoni M, Lobotesis K, Abu-Rumeileh S, Bagatto D, Mansoor N, Gigli GL, Valente M, Banerjee S (2024 May) Predictors of futile recanalization in nonagenarians treated with mechanical thrombectomy: a multi-center observational study. J Neurol 16. 10.1007/s00415-024-12428-8. Epub ahead of print. PMID: 38753228 10.1007/s00415-024-12428-8 PMC11319431 38753228

[52] Marios-Nikos P, Alex B, Jens F, Isabel F, Jan G, Mira K, Ronen L, Paolo M, Marc R, Jeffrey LS, Daniel S, Adriaan VE, Claus Z, Nikki R, Luzia B, Urs F (2024) EnDovascular Therapy Plus Best Medical Treatment (BMT) Versus BMT alone for MedIum distal VeSsel occlusion sTroke (DISTAL): an international, multicentre, randomized-controlled, two-arm, assessor-blinded trial.Eur Stroke J 2024 May 3:23969873241250212. 10.1177/23969873241250212. Epub ahead of print. PMID: 38702876. 10.1177/23969873241250212 PMC11569446 38702876

[53] Alexandre AM, Monforte M, Brunetti V, Scarcia L, Cirillo L, Zini A, Scala I, Nardelli V, Arbia F, Arbia G, Frisullo G, Kalsoum E, Camilli A, De Leoni D, Colò F, Abruzzese S, Piano M, Rollo C, Macera A, Ruggiero M, Lafe E, Gabrieli JD, Cester G, Limbucci N, Arba F, Ferretti S, Da Ros V, Bellini L, Salsano G, Mavilio N, Russo R, Bergui M, Caragliano AA, Vinci SL, Romano DG, Frauenfelder G, Semeraro V, Ganimede MP, Lozupone E, Romi A, Cavallini A, Milonia L, Muto M, Candelaresi P, Calabresi P, Pedicelli A, Broccolini A (2024) Baseline clinical and neuroradiological predictors of outcome in patients with large ischemic core undergoing mechanical thrombectomy: a retrospective multicenter study. Int J Stroke 19(7):779–788. 10.1177/17474930241245828Epub 2024 Apr 16. PMID: 38546177; PMCID: PMC11298113 38546177 10.1177/17474930241245828 PMC11298113

[54] Salim H, Musmar B, Adeeb N, Yedavalli V, Lakhani D, Grewal SS, El Naamani K, Henninger N, Sundararajan SH, Kühn AL, Khalife J, Ghozy S, Scarcia L, Tan BY, Regenhardt RW, Heit JJ, Cancelliere NM, Bernstock JD, Rouchaud A, Fiehler J, Sheth S, Puri AS, Dyzmann C, Colasurdo M, Barreau X, Renieri L, Filipe JP, Harker P, Radu RA, Abdalkader M, Klein P, Marotta TR, Spears J, Ota T, Mowla A, Jabbour P, Biswas A, Clarençon F, Siegler JE, Nguyen TN, Varela R, Baker A, Essibayi MA, Altschul D, Gonzalez NR, Möhlenbruch MA, Costalat V, Gory B, Stracke CP, Aziz-Sultan MA, Hecker C, Shaikh H, Liebeskind DS, Pedicelli A, Alexandre AM, Tancredi I, Faizy TD, Kalsoum E, Lubicz B, Patel AB, Pereira VM, Guenego A, Dmytriw AA (2024 May) MAD MT investigators. Outcomes of mechanical thrombectomy in anticoagulated patients with acute distal and medium vessel stroke. Eur Stroke J 10:23969873241249295. 10.1177/23969873241249295Epub ahead of print. PMID: 38726983 10.1177/23969873241249295 PMC11569456 38726983

[55] Scarcia L, Colò F, Alexandre AM, Brunetti V, Pedicelli A, Arba F, Ruggiero M, Piano M, Gabrieli JD, Ros VD, Romano DG, Cavallini A, Salsano G, Panni P, Limbucci N, Caragliano AA, Russo R, Bigliardi G, Milonia L, Semeraro V, Lozupone E, Cirillo L, Clarençon F, Zini A, Broccolini A emergent Carotid Artery Stenting (eCAS) study group. Effects of emergent carotid stenting performed before or after mechanical thrombectomy in the endovascular management of patients with tandem lesion: a multicenter retrospective matched analysis. AJNR Am J Neuroradiol. 2024 Jul 29:ajnr.A8421. 10.3174/ajnr.A8421. Epub ahead of print. PMID: 39025636 10.3174/ajnr.A8421 PMC11735429 39025636

[56] Consoli A, Pileggi M, Hasan AH, Rahman MH, Venier A, Sgreccia A, Pizzuto S, Coskun O, Di Maria F, Scarcia L, Lapergue B, Rodesch G, Bracard S, Chen B Unfavorable clinical outcomes in patients with good collateral scores following endovascular treatment for acute ischemic stroke of the anterior circulation: the UNCLOSE study. Interv Neuroradiol 2023 Nov 7:15910199231212519. 10.1177/15910199231212519. Epub ahead of print. PMID: 37936414. 10.1177/15910199231212519 PMC13294582 37936414

[57] Yedavalli VS, Salim HA, Musmar B, Adeeb N, Essibayi MA, ElNaamani K, Henninger N, Sundararajan SH, Kuhn AL, Khalife J, Ghozy S, Scarcia L, Tan BY, Heit JJ, Regenhardt RW, Cancelliere NM, Bernstock JD, Rouchaud A, Fiehler J, Sheth SA, Puri AS, Dyzmann C, Colasurdo M, Barreau X, Renieri L, Filipe JP, Harker P, Radu RA, Marotta TR, Spears J, Ota T, Mowla A, Jabbour P, Biswas A, Clarençon F, Siegler JE, Nguyen TN, Varela R, Baker A, Altschul D, Gonzalez N, Möhlenbruch MA, Costalat V, Gory B, Stracke P, Aziz-Sultan MA, Hecker C, Shaikh H, Liebeskind DS, Pedicelli A, Alexandre AM, Tancredi I, Faizy TD, Kalsoum E, Lubicz B, Patel AB, Mendes Pereira V, Guenego A, Dmytriw AA (2024) MAD MT Investigators. Symptomatic intracerebral hemorrhage in proximal and distal medium middle cerebral artery occlusion patients treated with mechanical thrombectomy. J Neurointerv Surg. Jul 24:jnis-2024-021879. 10.1136/jnis-2024-021879. Epub ahead of print. PMID: 38977305 10.1136/jnis-2024-021879 38977305

[58] Alexandre AM, Scarcia L, Brunetti V, Scala I, Kalsoum E, Valente I, Camilli A, De Leoni D, Colò F, Frisullo G, Piano M, Rollo C, Macera A, Ruggiero M, Lafe E, Gabrieli JD, Cester G, Limbucci N, Arba F, Ferretti S, Da Ros V, Bellini L, Salsano G, Mavilio N, Russo R, Bergui M, Caragliano AA, Vinci SL, Romano DG, Frauenfelder G, Semeraro V, Ganimede MP, Lozupone E, Romi A, Cavallini A, Milonia L, Muto M, Giordano F, Cirillo L, Calabresi P, Pedicelli A, Broccolini A (2023) Predictors of parenchymal hematoma and clinical outcome after mechanical thrombectomy in patients with large ischemic core due to large vessel occlusion: a retrospective multicenter study. J Neurointerv Surg. Dec 21:jnis-2023-021146. 10.1136/jnis-2023-021146. Epub ahead of print. PMID: 38129110 10.1136/jnis-2023-021146 38129110

[59] Sallustio F, Nicolini E, Saia V, Pracucci G, Mascolo AP, Marrama F, Gandini R, Da Ros V, Diomedi M, Alemseged F, Casetta I, Fainardi E, Castellan L, Del Sette M, Limbucci N, Nencini P, Bergui M, Cerrato P, Saletti A, De Vito A, Cioni S, Tassi R, Simonetti L, Zini A, Ruggiero M, Longoni M, Tessitore A, Ferraù L, Cavasin N, Critelli A, Vallone S, Bigliardi G, Zimatore DS, Petruzzellis M, Boghi A, Naldi A, Comai A, Dall’Ora E, Sanfilippo G, Persico A, Gallesio I, Sepe F, Menozzi R, Pezzini A, Besana M, Giossi A, Sanna A, Tassinari T, Burdi N, Boero G, Augelli R, Cappellari M, Cosottini M, Giannini N, Romano DG, Frauenfelder G, Nuzzi PN, Spinelli MC, Paladini A, Rizzo A, Filizzolo M, Mannino M, Timpani C, De Santis F, Carità G, Russo M, Galvano G, Sicurella L, Mangiafico S, Toni D (2024 Jun) Italian Registry of Endovascular Treatment in Acute Stroke (IRETAS) collaborators. Association between procedural time and outcome in unsuccessful mechanical thrombectomy for acute ischemic stroke: analysis from the Italian Registry of Endovascular Treatment in Acute Stroke. J Neurol 5. 10.1007/s00415-024-12458-2Epub ahead of print. PMID: 38836906 10.1007/s00415-024-12458-2 38836906

